# TRPA1-dependent reversible opening of tight junction by natural compounds with an α,β-unsaturated moiety and capsaicin

**DOI:** 10.1038/s41598-018-20526-7

**Published:** 2018-02-02

**Authors:** Yusuke Kanda, Youhei Yamasaki, Yoshie Sasaki-Yamaguchi, Noriko Ida-Koga, Shinji Kamisuki, Fumio Sugawara, Yoko Nagumo, Takeo Usui

**Affiliations:** 1 Graduate School of Life and Environmental Sciences, University of Tsukuba, 1-1-1 Tennodai, Tsukuba, Ibaraki 305-8572 Japan; 2 Department of Applied Biological Science, Faculty of Science and Technology, Tokyo University of Science, 2641 Yamazaki, Noda, Chiba 278-8510 Japan; 3 School of Veterinary Medicine, Azabu University, 1–17–71 Fuchinobe, Chuo-ku, Sagamihara, Kanagawa 252–5201 Japan; 4 Faculty of Life and Environmental Sciences, University of Tsukuba, 1-1-1 Tennodai, Tsukuba, Ibaraki 305-8572 Japan

## Abstract

The delivery of hydrophilic macromolecules runs into difficulties such as penetration of the cell membrane lipid bilayer. Our prior experiment demonstrated that capsaicin induces the reversible opening of tight junctions (TJs) and enhances the delivery of hydrophilic macromolecules through a paracellular route. Herein, we screened paracellular permeability enhancers other than capsaicin. As TJ opening by capsaicin is associated with Ca^2+^ influx, we first screened the compounds that induce Ca^2+^ influx in layered MDCK II cells, and then we determined the compounds’ abilities to open TJs. Our results identified several natural compounds with α,β-unsaturated moiety. A structure-activity relationship (SAR) analysis and the results of pretreatment with reducing reagent DTT suggested the importance of α,β-unsaturated moiety. We also examined the underlying mechanisms, and our findings suggest that the actin reorganization seen in capsaicin treatment is important for the reversibility of TJ opening. Furthermore, our analyses revealed that TRPA1 is involved in the Ca^2+^ influx and TJ permeability increase not only by an α,β-unsaturated compound but also by capsaicin. Our results indicate that the α,β-unsaturated moiety can be a potent pharmacophore for TJ opening.

## Introduction

There is an increasing need for biotechnological therapeutics (biologics) such as peptides, proteins, antibodies, polynucleotides and other macromolecular drugs, as they can act with high specificity and potency. Although recent progress in biotechnology has accelerated the processes for producing biologics on a commercial scale, improvements that address the biologics’ low oral bioavailability have been lacking. Hydrophilic macromolecular drugs are usually classified into class III of Biopharmaceutics Classification System (BSC), and for their administration, injections (i.e., by intravenous, intramuscular or subcutaneous routes) remain the most common routes.

However, injections decrease the patients’ quality of life. Non-invasive routes such as oral, nasal, pulmonary, transdermal and buccal routes are more favorable than injections, but their use also encounters several obstacles^1–3^. One of the most important of which is the epithelium, across which hydrophilic macromolecular drugs must move in order to exert their therapeutic effects^4^. Thus, the enhancement of the paracellular delivery of hydrophilic macromolecular drugs has received considerable attention^5^.

The paracellular movement of solutes and macromolecules across the epithelium is restricted by tight junctions (TJs). TJs are located at the apicolateral plasma membranes of adjacent cells^6^ and are composed of a complex combination of transmembrane integral proteins including occludin, claudins and tricellulin, along with several intracellular proteins such as zonula occludens-1 (Zo-1), which connects the transmembrane proteins to the actin cytoskeleton^7^. Therefore, TJ proteins and actin are key regulators of the TJs^8^. The opening of TJs by paracellular permeability enhancers (PPEs) is a way to increase the absorption of hydrophilic drugs across the epithelium^9,10^. In particular, reversible opening of TJ could allow such drugs to be safely and controllably absorbed. This approach is attractive because it could be applied to many different hydrophilic drugs, whereas the chemical modification of biologics to improve bioavailability requires full drug development for each compound^11^.

**Figure 1:**
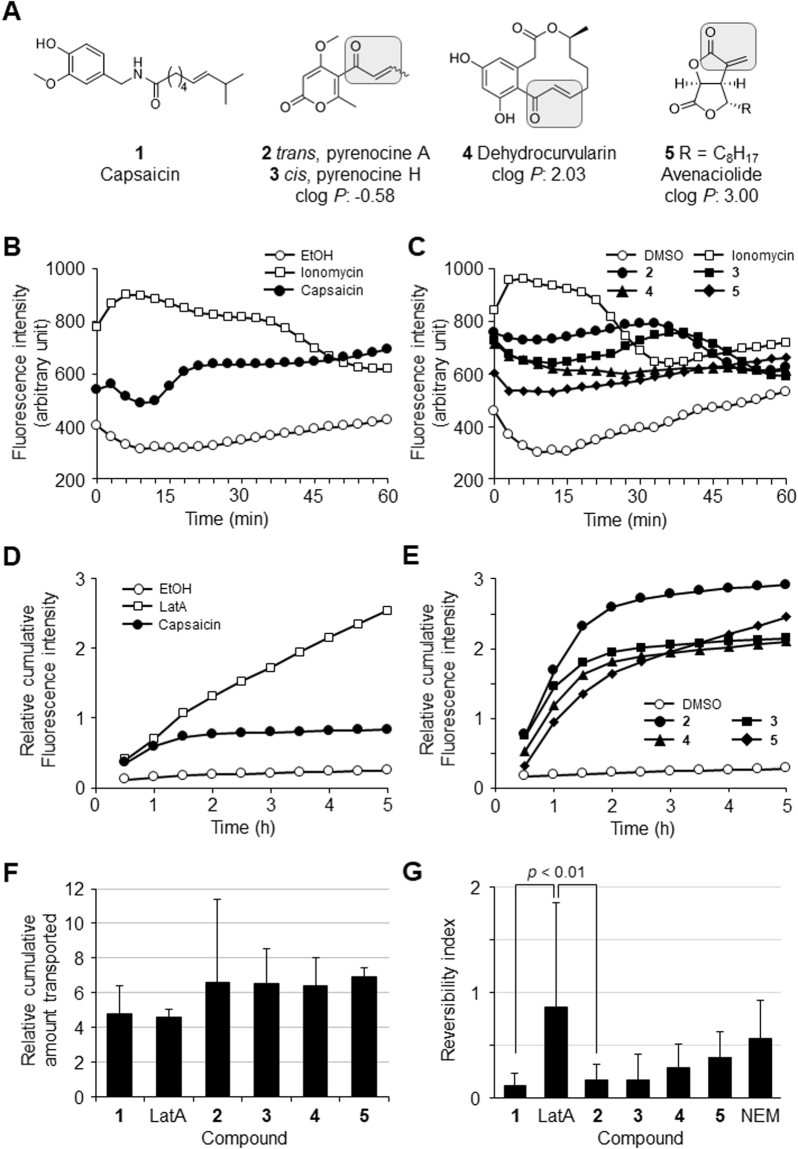
Discovery of four natural compounds that increase TJ permeability reversibly by a Ca^2+^ influx assay in the MDCK II cell monolayer. (**A**) The structures of capsaicin (**1**) and the screened compounds containing an α,β-unsaturated moiety identified in this study: pyrenocine A (**2**), pyrenocine H (**3**), dehydrocurvularin (**4**), and avenaciolide (**5**). α,β-unsaturated moiety is enclosed. The theoretical clog *P*-values of the four compounds are also shown. (**B**) Capsaicin induced a Ca^2+^ influx in the MDCK II monolayer. ○: EtOH. □: 10 μM ionomycin. ●: 300 μM capsaicin. Typical data of three independent experiments is shown. (**C**) Four hit compounds induced a Ca^2+^ influx in the MDCK II monolayer. ○: DMSO. □: 10 μM ionomycin. ●: 30 μM compound **2**. ■: 30 μM compound **3**. ▲: 10 μM compound **4**. ◆: 10 μM compound **5**. Typical data of three independent experiments is shown. (**D**) Capsaicin reversibly increased the paracellular permeability in the MDCK II monolayers, whereas LatA irreversibly increased it. ○: EtOH. □: 0.1 μM LatA. ●: 300 μM capsaicin. Typical data of three independent experiments is shown. (**E**) The four hit compounds reversibly increased the paracellular permeability in the MDCK II monolayer. ○: DMSO. ●: 30 μM compound **2**. ■: 30 μM compound **3**. ▲: 10 μM compound **4**. ◆: 10 μM compound **5**. Typical data of three independent experiments is shown. (**F**) The efficiencies of TJ permeability enhancement induced by the compounds were evaluated by the cumulative FD4 amounts transported until 2 h relative to vehicle control. Each value represents the mean ± S.D. of three independent experiments. (**G**) Reversibilities of TJ permeability induced by the compounds were evaluated by reversibility indexes (RIs). The results of ≥3 independent experiments were used to calculate each compound’s RI. *p*-values were calculated by Tukey’s multiple comparison test.

Capsaicin (structure shown in Fig. 1A, **1**), is a naturally occurring alkaloid that is responsible for a hot and spicy taste^12^. In previous research we demonstrated that capsaicin induces reversible opening of TJ, and we investigated the underlying mechanisms^13–15^. We observed that capsaicin treatment induced a Ca^2+^ influx that resulted in cofilin activation, accompanied by an F-actin alteration unique to capsaicin; in addition, F-actin decreased at bicellular junctions but increased at tricellular junctions. No change in TJ protein localization was observed upon exposure to capsaicin, but the amount of occludin was significantly decreased. Both the cofilin activation and occludin decrease contributed to the TJ opening by capsaicin. Those results suggest that capsaicin is a new type of PPE with unique mechanisms^16^.

Capsaicin is known as an agonist of TRPV1, a member of transient receptor potential (TRP) family of ion channels, which transmits noxious stimuli^17^. This suggests that the pungency of capsaicin could be a problem in the development of capsaicin as a PPE^18,19^. Although the question of whether capsaicin-induced TJ opening is dependent on TRPV1 is under investigation^20–22^, it may be helpful to have other PPEs with chemical structures different from that of capsaicin.

**Figure 2:**
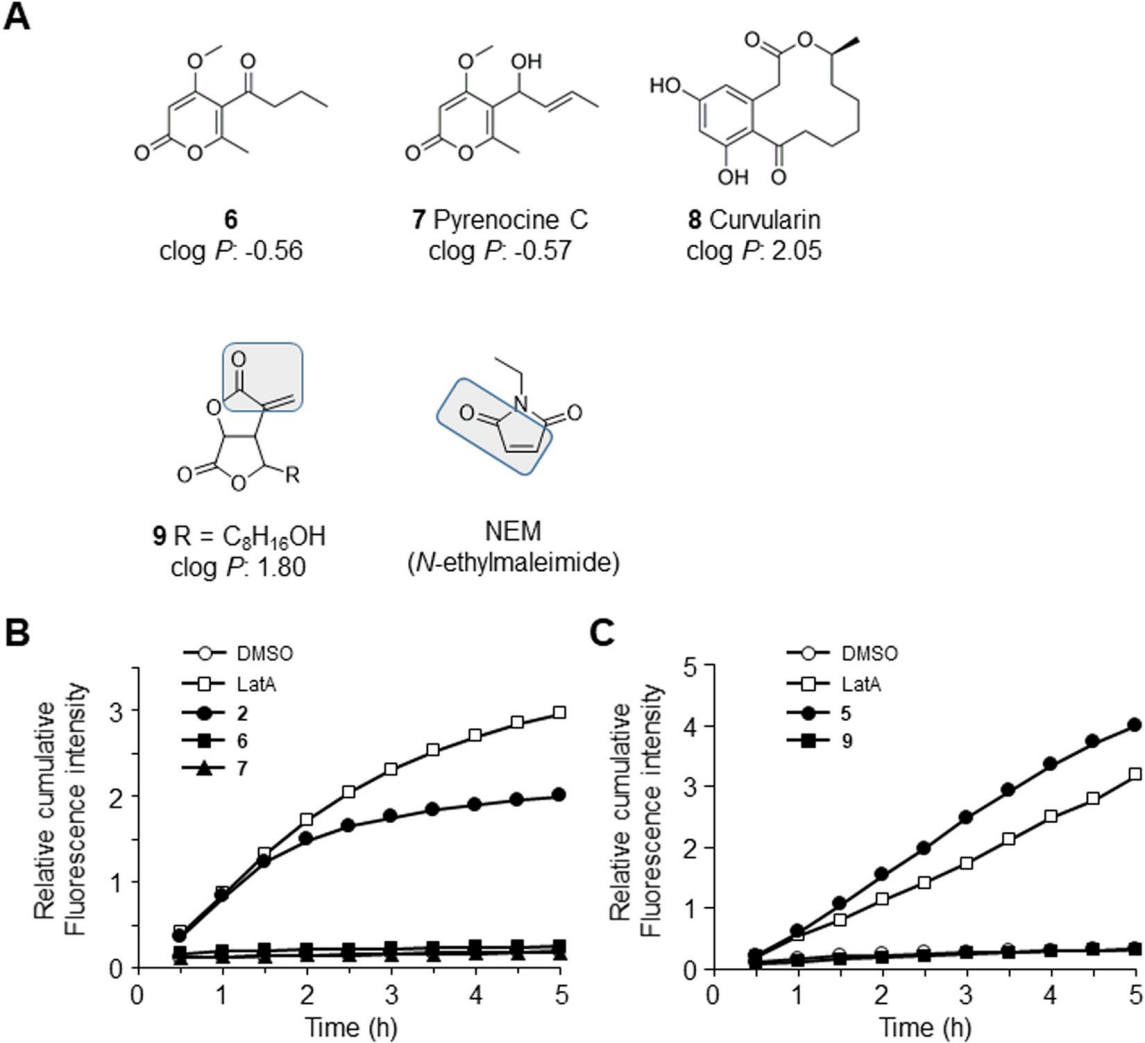
SAR analysis of four natural α,β-unsaturated compounds with TJ opening ability. (**A**) The structures of compounds **6**–**9** and NEM are shown. The compounds **6** and **7** are analogs of compounds **2** and **3**. Compounds **8** and **9** are analogs of compound **4** and compound **5**, respectively. α,β-unsaturated moiety of compounds **9** and NEM is enclosed. The theoretical clog *P-*values of compounds **6**–**9** are also shown. (**B**) The structural analogs that have no α,β-unsaturated carbonyl group: compounds **6** and **7** did not increase paracellular permeability in MDCK II monolayers. ○: DMSO. □: 0.1 μM LatA: ●: 30 μM compound **2** ■: 30 μM compound **6**. ▲: 30 μM compound **7**. Typical data of three independent experiments is shown. (**C**) The structural analog of compound **5**: compound **9** did not increase paracellular permeability in MDCK II monolayers, although it has an α,β-unsaturated carbonyl group. ○: DMSO. □: 0.1 μM LatA. ●: 10 μM compound **5**. ■: 10 μM compound **9**. Typical data of three independent experiments is shown.

TRPA1 is the other member of TRP family of ion channels which transmits a variety of noxious stimuli. Different from TRPV1, TRPA1 is activated by several cysteine-targeted compounds containing electrophiles through covalent binding at cysteine residues. Here we screened for TJ openers by conducting a Ca^2+^ influx and FD4 permeability assay of a Madin-Darby canine kidney (MDCK) II cell monolayer. The screening identified several natural compounds, a few of which have an α,β-unsaturated moiety: pyrenocine A (Fig. 1A, **2**)^23,24^, pyrenocine H (**3**), dehydrocurvularin (**4**)^25^, and avenaciolide (**5**)^26^. Further investigation of each compound’s ability to increase TJs’ permeability, including the role of α,β-unsaturated moiety and the underlying mechanisms affecting TJ integrity revealed that the TRPA1 channel is involved in the reversible TJ opening. Our present findings may lead to new PPE research and development.

## Results

### Screening using an MDCK II monolayer identified natural compounds with an α,β-unsaturated moiety as reversible permeability enhancers

In our previous research, we demonstrated that capsaicin (Fig. 1A, **1**) induces reversible TJ opening associated with a Ca^2+^ influx, cofilin activation and occludin decrease. We speculated that the Ca^2+^ influx is one of the essential events to induce TJ opening, based on the following observations: the short treatment of Ca^2+^ chelator EGTA can inhibit both cofilin activation and the associated decrease in transepithelial electrical resistance (TER)^13^, and blocking Ca^2+^ signaling by trifluoperazine also inhibited a capsaicin-induced cofilin activation, capsaicin-specific actin alteration, and the decrease in TER^15^.

In the present study we therefore screened 795 natural and synthetic compounds for TJ openers by performing a Ca^2+^ influx assay in an MDCK II cell monolayer. As shown in Fig. 1B, a sudden Ca^2+^ influx was induced by a positive control (ionomycin) and capsaicin in the MDCK II monolayer. The Ca^2+^ indicator fluorescence by ionomycin decreased at approx. 20 min, but that by capsaicin was sustained until at least 60 min.

Using this Ca^2+^ influx assay system, we identified several natural and synthetic compounds. Interestingly, among the hit compounds, four natural compounds, pyrenocine A (**2**), pyrenocine H (**3**), dehydrocurvularin (**4**), and avenaciolide (**5**) have a common structural feature: an α,β-unsaturated moiety (Fig. 1A). The Ca^2+^ influx induced by these four compounds was quick and relatively sustained (Fig. 1C).

We conducted a secondary screening to evaluate these compounds’ abilities to increase TJ permeability. FITC-dextran-4 (FD4) solution with each compound was applied to the apical side of the MDCK II monolayer in transwells, and basolateral solution was collected every 30 min to measure the permeability. Figure 1D shows the cumulative transports induced by capsaicin and latrunculin A (LatA). Capsaicin induced an FD4 permeability increase as quickly as 30 min and until 2 hr, and then spontaneously stopped (without the removal of capsaicin), indicating a reversible opening of TJ. In contrast, LatA, which opens TJ irreversibly^15^, increased the FD4 permeability continuously. All four α,β-unsaturated compounds enhanced the permeability from 30 min, the beginning of the measurement, although there were differences in the compounds’ efficiency and reversibility (Fig. 1E).

Therefore, we next analyzed the reversibility of TJ opening induced by each compound. Although the apparent permeability coefficient is usually used for the evaluation of the penetration-enhancing effects of an agent^27,28^, this coefficient is not applicable for our compounds because of their reversible pattern. We thus chose to evaluate how much larger amounts they transported cumulatively until 2 h, compared to the control (Fig. 1F). All four α,β-unsaturated compounds were similarly effective to transport FD4.

Next, we evaluated the compounds’ reversibility by calculating the reversibility index as the slope between 4.5 h and 5.0 h divided by the slope between 0.5 h and 1.0 h in the FD4 cumulative transport results (Fig. 1G). In the case of capsaicin, a reversible permeability enhancer, the slope between 0.5 h and 1.0 h was larger than that between 4.5 h and 5.0 h, because capsaicin enhanced the permeability considerably between 0.5 h and 1.0 h, but not between 4.5 h and 5.0 h, resulting in a low index value (0.12 in Fig. 1G). In contrast, LatA, an irreversible permeability enhancer, maintained similar permeability enhancement between the two durations, giving a high value (0.85). For compound **2**, the index value was calculated as 0.16. The statistical difference between the reversibility indexes of compound **2** and capsaicin was not <0.05, whereas the difference between those of compound **2** and LatA is <0.01. This suggests that the permeability enhancement by compound **2** is reversible similarly to that by capsaicin. The reversible opening of TJ by compound **2** was also confirmed by TER measurement (Suppl Fig. S1). Compounds **3**–**5** have the reversibility indices of 0.17, 0.29, and 0.37, respectively, and the statistical difference was not significant with both capsaicin and LatA, suggesting that these compounds are partial reversible permeability enhancers.

### The structure-activity relationship (SAR) results show the importance of the α,β-unsaturated moiety for TJ opening

As shown above, we demonstrated that all four hit compounds induced a Ca^2+^ influx and reversible/partial reversible opening of TJ in an MDCK II monolayer. Each of the compounds has an α,β-unsaturated moiety in common, but other structural features are completely different: α,β-unsaturated ketone or ester, with or without a resonance structure, bicyclic or not, ring size variety, etc. Even when focusing on carbon-carbon double bonds in the α,β-unsaturated moiety, the compounds vary: e.g., acyclic double bonds with *cis* and *trans* configurations, and cyclic or exocyclic double bonds. We therefore examined the importance of the α,β-unsaturated moiety on TJ permeability increase by conducting a structure-activity relationship (SAR) analysis.

As shown in Fig. 2B, we analyzed whether compound **6** (Fig. 2A), which is an analog of compounds **2** and **3** with only differences in the α and β positions saturated, would enhance FD4 permeability. The results revealed that compound **6** did not increase the permeability at all in the conditions in which compound **2** clearly increased it. Compound **7** (pyrenocine C in Fig. 2A)^29^, in which the α,β-unsaturated ketone is reduced to alcohol, also did not enhance the permeability. We next analyzed an analog of compound **4**: compound **8**^25^ with only differences in the α and β positions saturated. Compound **8** hardly enhanced the permeability compared to compound **4** (Suppl. Fig. S2). In contrast, compound **9**, an analog of compound **5** which keeps the α,β-unsaturated moiety and has a hydroxyl moiety at the terminal of an aliphatic side chain of compound **5**, did not induce TJ opening (Fig. 2C). These data indicate that the α,β-unsaturated moiety is important but not sufficient to open TJs. Of note, the structural analogs without TJ-opening ability, i.e., compounds **6**–**9** did not induce a Ca^2+^ influx (Suppl. Fig. S3).

To investigate other structural factors that may be required for TJ-opening ability, we estimated lipophilicity differences between each hit compound and its analogs, by calculating theoretical partition coefficients (clog *P*) (Figs 1A, 2A). Compounds **2** and **3** had the lowest clog *P*-value: −0.58. For their analogs compounds **6** and **7**, the clog *P* was −0.56 and −0.57, respectively; there is only a marginal difference between compound **2** and **3** and their analogs compound **6** and **7**. This is also the case with the difference between compound **4** (clog *P*: 2.03) and **8** (clog *P*: 2.05). These data suggest that compounds **6**, **7**, and **8** lost the Ca^2+^ influx and the permeability enhancement ability not because of their lipophilicity change, but rather because of the loss of the α,β-unsaturated moiety. In the case of compound **5**, it has the highest clog *P*-value: 3.00. Notably, the difference between compound **5** and its analog compound **9** (clog *P*: 1.80) was found to be larger. This lipophilicity change can be one of the reasons why compound **9** is unable to induce TJ opening, even though compound **9** keeps an α,β-unsaturated moiety.

### The involvement of the α,β-unsaturated moiety in the opening of TJ suggests that an α,β-unsaturated moiety can be a potent pharmacophore for a PPE

**Figure 3:**
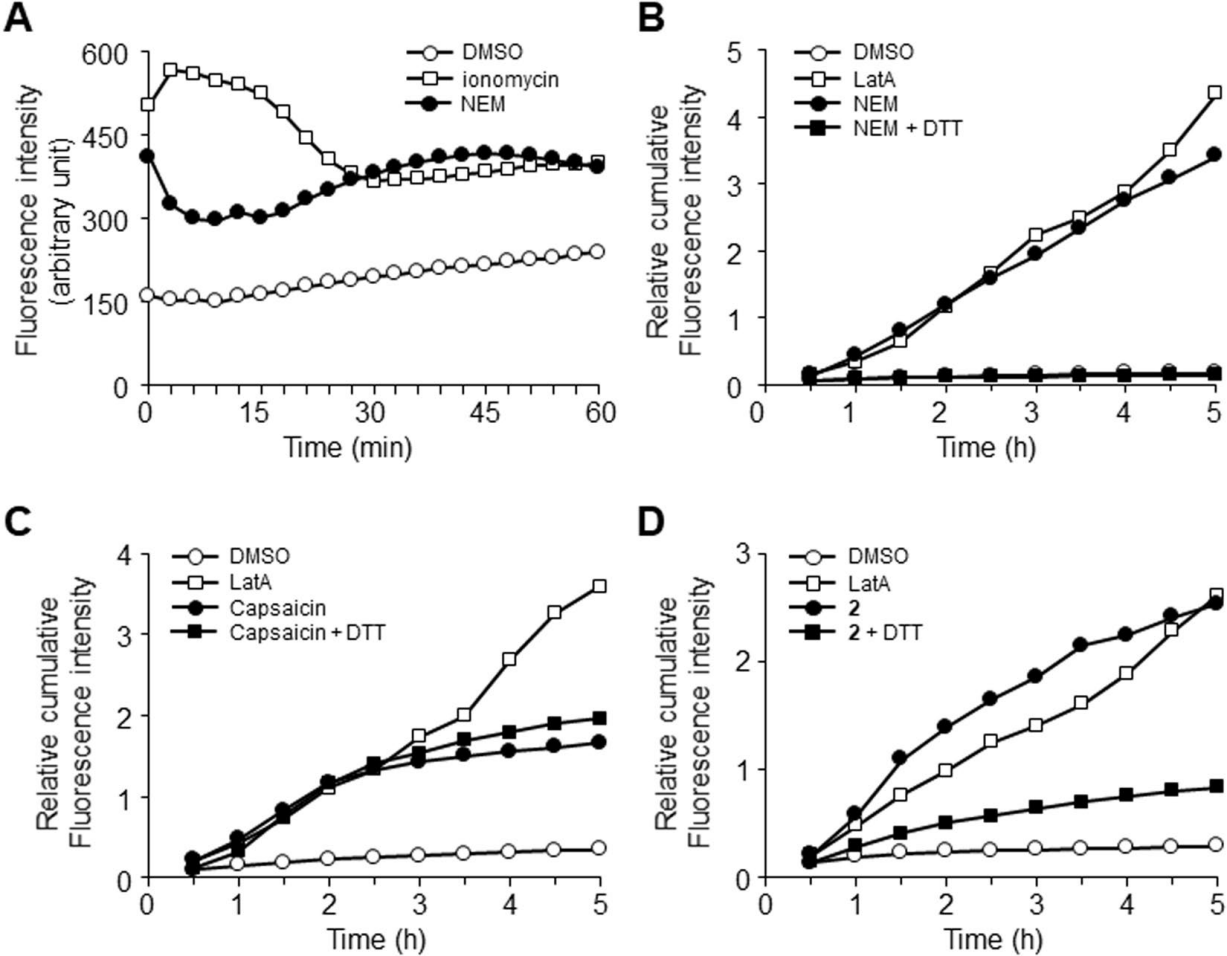
Involvement of the α,β-unsaturated moiety as a potent pharmacophore to open TJs. (**A**) NEM induced Ca^2+^ influx in MDCK II monolayers. ○: DMSO. □: 0.1 μM LatA. ●: 30 μM NEM. Typical data of three independent experiments is shown. (**B**) NEM increased the paracellular permeability in the MDCK II monolayer, but DTT pretreatment for 10 min abolished the permeability increase. ○: DMSO, □: 0.1 μM LatA, ●: 30 μM NEM, and ■: 30 μM NEM pretreated with 30 μM DTT. Typical data of three independent experiments is shown. (**C**) DTT pretreatment for 10 min did not affect the capsaicin-induced TJ opening. ○: EtOH, □: 0.1 μM LatA, ●: 300 μM capsaicin, and ■: 300 μM capsaicin pretreated with 300 μM DTT. Typical data of three independent experiments is shown. (**D**) DTT pretreatment abolished the permeability increase induced by compound **2**. ○: DMSO, □: 0.1 μM Lat A, ●: 30 μM compound **2**, and ■: 30 μM compound **2** pretreated with 30 μM DTT. Typical data of five independent experiments is shown.

To further confirm the importance of the α,β-unsaturated moiety, we investigated whether or not a representative α,β-unsaturated small molecule, *N*-ethylmaleimide (Fig. 2A, NEM), induces TJ opening. First, we checked NEM’s ability to induce a Ca^2+^ influx. NEM also showed a quick influx and sustained Ca^2+^ indicator fluorescence (Fig. 3A). Next we checked the FD4 permeability increase by NEM. Figure 3B shows that NEM opened TJs. The reversibility index of NEM was 0.56, with no significant difference compared to both those of capsaicin and LatA (Fig. 1G). This suggests that the permeability enhancement by NEM is also partially reversible similarly to that of compound **3**–**5**.

We also analyzed the importance of α,β-unsaturated moieties in TJ opening by *in situ* modification of the moiety. The thiol moiety of dithiothreitol (DTT) can be added to the β-carbon of α,β-unsaturated carbonyl compounds, a mechanism referred to as the Michael reaction. DTT pretreatment of NEM completely abolished NEM’s TJ-opening ability (Fig. 3B), whereas the pretreatment of capsaicin with DTT did not affect at all (Fig. 3C). As shown in Fig. 2D, DTT also considerably inhibited TJ opening by compound **2**. Similarly, compounds **3**, **4**, and **5** lost its ability to open TJ by pretreatment with DTT (Suppl. Fig. S4).

The results of the above analyses taken together suggest that the α,β-unsaturated moiety plays an indispensable role in the Ca^2+^ influx and permeability enhancement by compounds **2**–**5** and NEM, but the moiety is not sufficient. This is the first finding about a relationship between α,β-unsaturated moieties and TJ opening, to the best of our knowledge. Our findings indicate that the α,β-unsaturated moiety can be a potent pharmacophore for a PPE.

### The mechanistic comparison of TJ opening by capsaicin and α,β-unsaturated compounds

**Figure 4:**
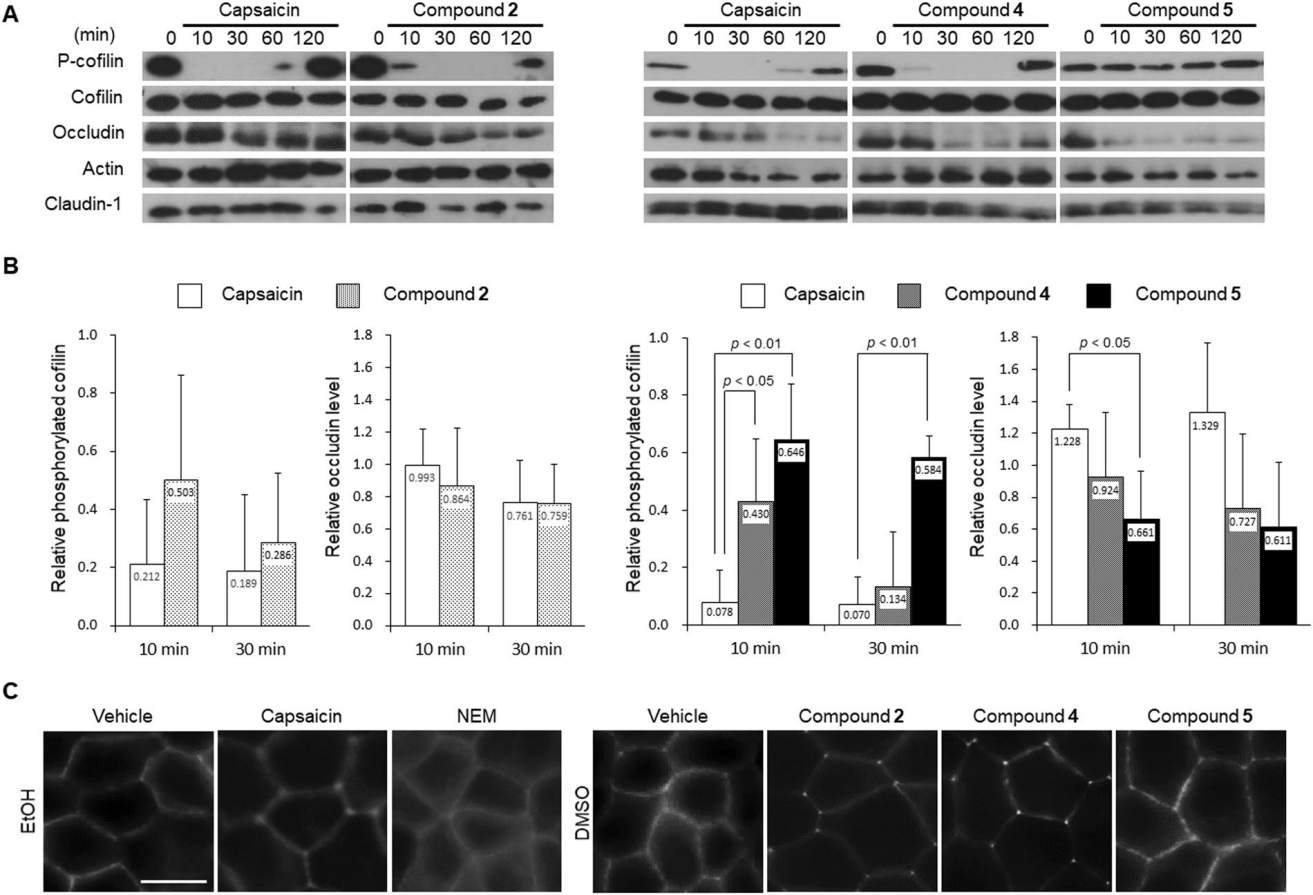
Mechanistic similarities/differences underlying the reversible opening of TJ between capsaicin and α,β-unsaturated compounds. (**A**) Western blot detection of cofilin phosphorylation, occludin and claudin-1. MDCK II monolayers were treated with (*left panel*) 300 μM capsaicin and 30 μM compound **2**, and (*right panel*) 300 μM capsaicin, 10 μM compounds **4** and **5** for indicated durations. Typical data of three independent experiments is shown. The samples of upper and lower panels derived from the same experiment and full length figures are presented in Suppl Fig. 6. (**B**) The densitometric analysis of phosphorylated cofilin (*left*) and occludin (*right*) from three independent experiments performed with NIH ImageJ software. The band intensity of each time point relative to that of time 0 are shown. Student’s *t-test* for compound **2** and Dunnett’s test for compounds **4** and **5** has been performed comparing each compound with capsaicin at each time point. (**C**) Compound **2** and **4** affected the distribution of actin filament similarly to capsaicin, but NEM and compound **5** did not. MDCK II monolayers were treated with 300 μM capsaicin, 30 μM compound **2** and NEM, 10 μM compounds **4** and **5** for 45 min. Scale bar: 10 μm. Typical data of three independent experiments is shown.

Our earlier research disclosed that capsaicin opens TJs reversibly through both cofilin activation resulting in actin reorganization, and a decrease in occludin. In the present study, to investigate the mechanisms of novel TJ permeability enhancers, we checked whether they show the same reactions. As we reported previously, capsaicin induced cofilin dephosphorylation (activation) within 10 min, and the phosphorylation recovery was observed after approx. 60 min (Fig. 4A). Compounds **2** and **4** induced cofilin dephosphorylation strongly and immediately, and it also recovered afterwards. In contrast, cofilin dephosphorylation by compound **5** was notably weaker.

We analyzed the extent of cofilin activation by capsaicin and three compounds by measuring the phosphorylation level of cofilin at 10 and 30 min, because cofilin activation ceased after 60 min. As shown in Fig. 4B, compound **4** showed a retardation of cofilin activation compared to capsaicin, but later on, there is no difference between its effect and that of capsaicin. At 30 min, compounds **2** and **4** reduced phosphorylated cofilin to 10–20% relative to that at time 0. Compound **5** retained cofilin phosphorylation compared to capsaicin at both 10 and 30 min. The maximum reduction in cofilin phosphorylation by compound **5** was observed at 30 min, and was as high as 50% at time 0.

We next checked the occludin decrease. Capsaicin decreased the occludin protein after about 1 h. Compounds **2**, **4**, and **5** also decreased occludin, although the extent of occludin decrease varied. Again, we analyzed the relative occludin content at 10 and 30 min after treatment, to observe early events of the TJ opening phase, not the recovery phase. The densitometric analysis showed that compounds **2**, **4**, and **5** seem to decrease occludin at 10 and 30 min, but not significantly compared to capsaicin (Fig. 4B). Claudin-1, another integral membrane protein composing TJs, showed no remarkable change by these compounds (Suppl. Fig. S5). We also could not find significant change of tricellulin, a tricellular protein involving macromolecule transport, in capsaicin and compound **2** treated cells (Suppl. Fig. S6).

As we observed that cofilin activation by compound **5** was significantly weak, we checked the actin reorganization induced by cofilin activation. The perijunctional actin structures were observed by F-actin staining in EtOH or DMSO vehicle treatment (Fig. 4D). Capsaicin reduced the level of perijunctional actin at bicellular junctions but produced actin-rich regions at tricellular junctions^15^. Compounds **2** and **4** exhibited the same phenomenon, whereas compound **5** and NEM did not. Because compound **5** and NEM had the least reversibility, actin reorganization and the efficiency of reversible opening of TJ might be related. The localization of claudin-1, occludin, Zo-1 and tricellulin were not changed by compound **2**, like capsaicin as previously we reported (Suppl. Fig. S7)^15^.

### Compound 2 and capsaicin open TJs via the activation of TRPA1

Earlier studies showed that structurally unrelated electrophilic compounds activated TRPA1 through the covalent modification of cysteine residues^30–32^. In addition, it was recently reported that the TRPA1 agonist allyisothiocyanate (AITC) reduced the TER of MDCK II cell layers, offering the TRPA1 channel as a regulator of epithelial cell barriers^33^. It is also noteworthy that the non-pungent capsaicin analog capsiate activates TRPA1^34^. We thus examined whether compound **2**, a representative electrophilic molecule among the four natural molecules we identified in this study, uses TRPA1 activation to open TJs.

**Figure 5:**
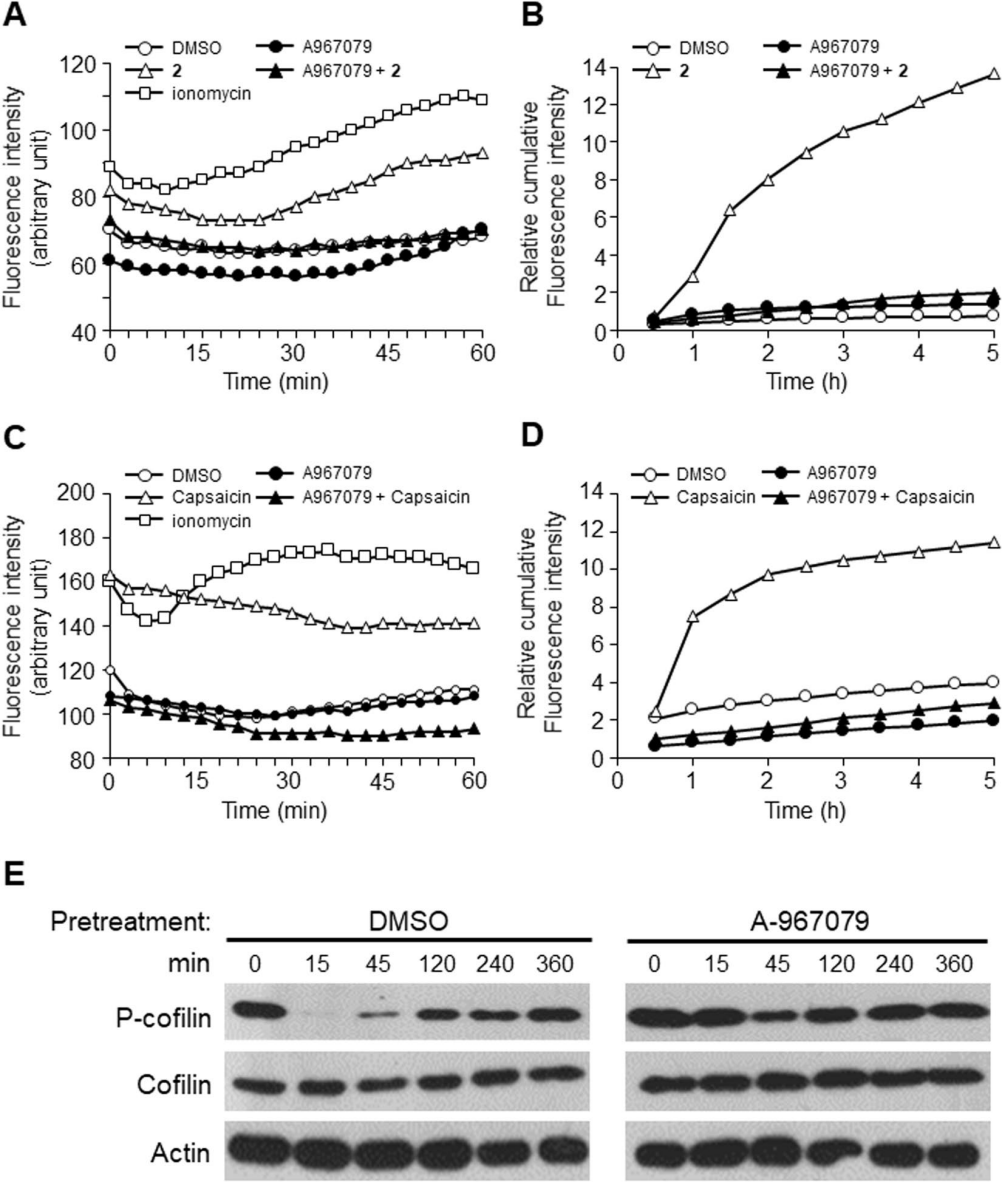
Compound **2** and capsaicin both induced a Ca^2+^ influx and a permeability increase via activation of TRPA1 in MDCK II monolayers. (**A**) TRPA1 antagonist A-967079 pretreatment for 30 min abolished the Ca^2+^ influx induced by compound **2** in the MDCK II monolayer. Typical data of three independent experiments is shown. ○: DMSO, □: 10 μM ionomycin, △: 30 μM compound **2**, ▲: 30 μM compound **2** pretreated with 1 μM A-967079, and ●: 1 μM A-967079. (**B**) TRPA1 antagonist A-967079 pretreatment for 30 min inhibited the permeability increase induced by compound **2** in MDCK II monolayer. Typical data of three independent experiments is shown. ○: DMSO, △: 30 μM compound **2**, ▲: 30 μM compound **2** pretreated with 1 μM A-967079, and ●: 1 μM A-967079. (**C**) TRPA1 antagonist A-967079 pretreatment for 30 min abolished the capsaicin-induced Ca^2+^ influx in the MDCK II monolayer. Typical data of three independent experiments is shown. ○: DMSO, □: 10 μM ionomycin, △: 300 μM capsaicin, ▲: 300 μM capsaicin pretreated with 1 μM A-967079, and ●: 1 μM A-967079. (**D**) TRPA1 antagonist A-967079 pretreatment for 30 min inhibited the capsaicin-induced permeability increase in the MDCK II monolayer. Typical data of three independent experiments is shown. ○: DMSO, △: 300 μM capsaicin, ▲: 300 μM capsaicin pretreated with 1 μM A-967079, ●: 1 μM A-967079. (**E**) Cofilin dephosphorylation induced by 300 μM capsaicin was inhibited by pretreatment with 1 μM A-967079 for 30 min. Typical data of three independent experiments is shown. The samples derived from the same experiment and full length figures are presented in Suppl. Fig. 7.

Because the activation of TRPA1 increases the influx of Ca^2+^, we first examined TRPA1’s contribution to the Ca^2+^ influx induced by compound **2** in an MDCK II monolayer. Pretreatment with the TRPA1 antagonist A-967079 before compound **2** greatly reduced the Ca^2+^ influx in the MDCK II monolayer (Fig. 5A). Next, we investigated whether the TRPA1 antagonist also affects the TJ permeability increase by compound **2** in the monolayer. Figure 5B clearly shows that A-967079 inhibits the TJ opening by compound **2**. These results strongly suggest that the α,β-unsaturated natural compound **2** activates TRPA1 to induce a Ca^2+^ influx and TJ permeability increase.

We then examined whether TRPA1 is also involved in the capsaicin-induced TJ permeability increase. Not only the Ca^2+^ influx (Fig. 5C) but also the FD4 permeability (Fig. 5D) induced by capsaicin were completely blocked by pretreatment with A-967079. We analyzed the effects of A-967079 on the cofilin dephosphorylation and actin reorganization induced by capsaicin, because they are expected to be regulated by Ca^2+^ signaling^13–15^. As shown in Fig. 5E, A-967079 inhibited the cofilin dephosphorylation. Actin reorganization was also attenuated by A-967079 pretreatment (Suppl. Fig. S8A).

**Figure 6:**
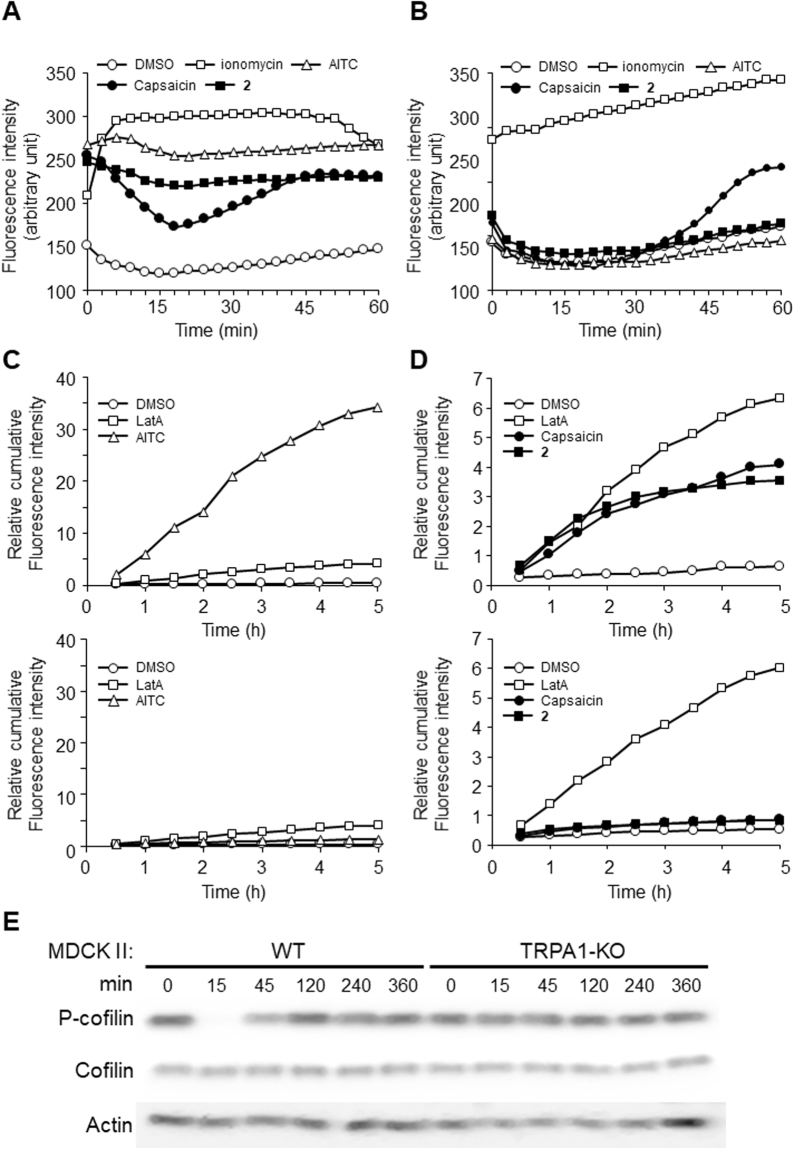
TRPA1 agonist AITC, compound **2** and capsaicin failed to induce TJ permeability increase in TRPA1-KO MDCK II monolayers. (**A**) TRPA1 agonist AITC induced a Ca^2+^ influx in the MDCK II monolayer, as did compound **2** and capsaicin. Typical data of three independent experiments is shown. ○: DMSO, □: 10 μM ionomycin, △: 30 μM AITC. ■: 30 μM compound **2**. ●: 300 μM capsaicin. Typical data of three independent experiments is shown. (**B**) TRPA1 agonist AITC failed to induce a Ca^2+^ influx in the TRPA1-KO MDCK II monolayer. Typical data of three independent experiments is shown. ○: DMSO, □: 10 μM ionomycin, △: 30 μM AITC. ■. 30 μM compound **2**. ●: 300 μM capsaicin. (**C**) TRPA1 agonist AITC increased the TJ permeability in the MDCK II monolayer but not in the TRPA1-KO monolayer. Typical data of three independent experiments is shown. *Upper graph*: WT MDCK II monolayer. *Lower graph*: TRPA1-KO MDCK II monolayer. ○: DMSO. □: 0.1 μM Lat A. △: 30 μM AITC. (**D**) The TJ permeability increases induced by both compound **2** and capsaicin in the MDCK II monolayer were abolished in the TRPA1-KO monolayer. Typical data of three independent experiments is shown. *Upper graph*: WT MDCK II monolayer. *Lower graph*: TRPA1-KO MDCK II monolayer. ○: DMSO. □: 0.1 μM Lat A. ■: 30 μM compound **2**. ●: 300 μM capsaicin. (**E**) Cofilin dephosphorylation was induced by 300 μM capsaicin in the WT MDCK II monolayer but not in the TRPA1-KO monolayer. The samples derived from the same experiment and full length figures are presented in Suppl Fig. 8.

Finally, we confirmed TRPA1 involvement using TRPA1-knockout (TRPA1-KO) MDCK II cells prepared by the CRISPR/Cas9 system. First, we checked the Ca^2+^ influx induced by TRPA1 agonist AITC in wild-type (WT) and TRPA1-KO MDCK II monolayers. Figure 6A shows that in the WT MDCK II monolayer, 30 μM AITC induced a significant Ca^2+^ influx compared to compound **2** and capsaicin. In contrast, in the TRPA1-KO monolayer, AITC completely lost its ability to induce a Ca^2+^ influx (Fig. 6B), verifying the TRPA1-KO. Of note, capsaicin still showed a Ca^2+^ influx at the last part of the measurement.

We then investigated whether an FD4 permeability increase is induced by AITC, and if so, whether it is affected by the existence of TRPA1. Figure 6C shows that in the WT monolayer, 30 μM AITC induced significant FD4 permeability (Fig. 6C, upper graph), and this was abolished completely in the TRPA1-KO monolayer (Fig. 6C, lower graph). These results suggest that AITC can increase the TJ permeability via TRPA1 activation, corresponding with the previous finding that 100 μM AITC reduced the TER of MDCK II cell layers^33^.

We next applied capsaicin and compound **2** to both the WT and TRPA1-KO MDCK II monolayers. As shown in Fig. 6D, the TJ permeability increases induced by capsaicin and compound **2** in the WT monolayers (Fig. 6D, upper graph) were not observed in the TRPA1-KO monolayers (Fig. 6D, lower graph), indicating that these two compounds also increase TJ permeability via the activation of TRPA1. Figure 6E and Supplementary Figure S8B confirmed that the cofilin dephosphorylation and actin reorganization induced by capsaicin were also abolished or attenuated in TRPA1-KO cells compared to WT cells.

Taken together, these results indicate that the α,β-unsaturated natural compounds we identified increase TJ permeability via TRPA1 activation, and that the induction of reversible TJ permeability by capsaicin is mediated via TRPA1.

## Discussion

In this study, we discovered α,β-unsaturated natural compounds with TJ-opening ability by conducting Ca^2+^ influx screening on MDCK II cell monolayers. This is apparently the first finding of a relationship between α,β-unsaturated moieties and TJ opening. In the range we examined here, the α,β-unsaturated compounds that increased TJ permeability, including NEM, also induced a Ca^2+^ influx. Therefore, Ca^2+^ influx might be a trigger of TJ opening by α,β-unsaturated compounds, as is the case for capsaicin. However, it is important to emphasize that not all the α,β-unsaturated compounds induce a Ca^2+^ influx and TJ opening. For example, compound **9** with an α,β-unsaturated moiety but no TJ-opening ability does not induce a Ca^2+^ influx (Suppl. Fig. S3). Therefore, α,β-unsaturated moieties is one of important but not the sufficient factor for TJ opening. The structure and activity relationships is summarized in Supplementary Table S1.

Our findings indicate that the α,β-unsaturated moiety could be a pharmacophore for TJ opening. However, the introduction of a hydroxyl group into the side chain of compound **5** resulting in compound **9** failed to open TJs (Fig. 2C), indicating that the α,β-unsaturated moiety is not sufficient to open TJs. The compound’s hydrophobicity is probably one of the other factors required for TJ opening (Figs 1A, 2A). Compound **9**, the analog of compound **5** with only an additional terminal hydroxyl group, has a considerably low clog *P*-value (1.80) compared to 3.00 of compound **5**. Because cysteine residues required for TRPA1 activation are located in cytoplasm side, it is required for compounds to pass through the plasma membrane to attack these cysteine residues of TRPA1. Hydrophilic property of compound **9** might decrease the ability to pass through the plasma membrane. Although further SAR analyses are required, these results suggest that the hydrophobicity of compound **5** can be important for the TJ-opening ability. In addition to an α,β-unsaturated moiety, each structure should have optimal hydrophobicity.

We observed that there are mechanistic similarities between capsaicin and the novel TJ permeability enhancer compound **2**: Ca^2+^ influx, cofilin activation, occludin decrease, and reversible opening of TJ. This is noteworthy because we demonstrated that both the cofilin activation and occludin decrease contribute to capsaicin’s reversible TJ opening ability^15^. Compound **5** showed weak cofilin activation, quicker occludin decrease, and low reversibility (Figs 1E,G, 4A,B). The weaker cofilin activation by compound **5** possibly resulted in the loss of actin reorganization (Fig. 3D), because not only cofilin regulates actin dynamics^35,36^; we also previously observed that cofilin activation and capsaicin-specific actin reorganization were correlated^15^. The fast occludin decrease in the absence of actin alteration might explain the low reversibility of compound **5**. In the previous study, we demonstrated that actin alteration and its recovery accompany capsaicin’s reversible TJ opening, suggesting that actin plays an important role in the reversibility. Because we did not observe substantial actin alteration by compound **5**, this compound must be more dependent on occludin decrease to open TJs, inducing a significantly quicker occludin decrease compared to that induced by capsaicin, resulting in low reversibility.

Perturbations of occludin are also known to increase TJ permeability^21,37–39^. Why are both actin alternation and an occludin decrease required to occur at the same time for reversible but not for substantial TJ opening? One possibility is that the modulation by either alone to substantially open TJs might be too severe, resulting in irreversible opening of TJ. This is supported by our present observation that complete actin severing by LatA resulted in irreversible opening of TJ (Fig. 1D,G).

The other possibility is that a collapse of the actin scaffold is required for the remodeling of the TJ complex from the occludin-containing form to the occludin-lacking form. It was reported that occludin is not an essential component for TJs^40^. Because the anchoring TJ complex on the cytoplasmic side by actin filaments is important for stabilizing the TJ complex structure, the collapse of actin filaments probably leads to a destabilization of the TJ complex and provides an opportunity for remodeling. In this regard, capsaicin opens TJ reversibly because capsaicin induces the combination of actin reversible modulation and an occludin decrease, but compound **5** and NEM are partial reversible TJ opener because these compounds fail to induce TJ remodeling. Further structural investigations are required to test this possibility.

We have observed that Ca^2+^ influx is important for TJ opening not only by capsaicin but also natural compounds with an α,β-unsaturated moiety studied here. Because an α,β-unsaturated moiety can bind to a molecular target covalently, these compounds might induce a Ca^2+^ influx via covalent binding to the target. A cation channel, TRPA1, is known to be activated by structurally unrelated electrophiles through the modification of cysteine residues within the cytoplasmic N terminus of the channel^30,31^. It was recently demonstrated that MDCK II cells express TRPA1, contributing to Ca^2+^ influx and TJ regulation^33^. As speculated, here we observed that compound **2** activates TRPA1 to induce a Ca^2+^ influx and TJ permeability increase.

We also discovered that not only compound **2** but also capsaicin activates TRPA1 to induce the Ca^2+^ influx and TJ permeability increase. Their structures are diverse and their chemical reactivities are also different with or without electrophilic functional group. Because capsaicin is an agonist of TRPV1 cation channel, its mode of action to TRPA1 is not clear^34,41–43^. It is reported that some compounds which lack of covalent reactivity, for example capsiate, activate TRPA1^34^. Therefore, there is a possibility that capsaicin also binds non-covalently and activates TRPA1 directly. Alternatively, capsaicin might activate TRPA1 indirectly via unknown receptor. There is also difference between TRPA1 agonist AITC and capsaicin, as capsaicin still induced small Ca^2+^ influx in TRPA1-KO cells. We are now investigating the precise activation and interaction mode.

In conclusion, we identified a new category of reversible openers for TJ. Four α,β-unsaturated natural compounds revealed by the screening can open TJs within 30 min. Aside from the α,β-unsaturated moiety that these four compounds have in common, the compounds have different structures and varying lipophilicity. This is the first report that an α,β-unsaturated moiety can serve as a potent pharmacophore to open TJs reversibly. We also analyzed the compounds’ underlying mechanisms, and our results suggest that the combination of actin alteration and occludin decrease might be important for reversible opening of TJ. TRPA1 was shown to be responsible for the Ca^2+^ influx and TJ permeability increase by not only compound **2** but also capsaicin. The present study disclosed the key structural factors and molecular target, and these findings will contribute to the design of new TJ permeable enhancers and to new PPE research and development.

## Methods

### Natural compounds isolation

Natural products were extracted essentially as described^44^. Culture broths of fungal strains isolated from seaweeds, mosses, and other plants were extracted with CH_2_Cl_2_. The crude extracts were separated by silica gel column chromatography to purify compounds. Compounds **2–5, 7** and **8** were identified by comparison of their reported ^1^H and ^13^C nuclear magnetic resonance (NMR) and mass spectroscopy (MS) data^29,45–48^. We determined the structures of compounds **6** and **9** by interpreting the spectroscopic data (1D/2D NMR and MS). Compound **6**: ^1^H NMR (400 MHz, CDCl_3_), δ 5.47 (s, 1H), 3.85 (s, 3H), 2.68 (t, *J* = 7.4 Hz, 2H), 2.23 (s, 3H), 1.66 (tq, *J* = 7.4, 7.4 Hz, 2H), 0.95 (t, *J* = 7.4 Hz, 3H); ^13^C NMR (100 MHz, CDCl_3_), δ 201.1, 168.4, 162.7, 162.4, 114.2, 87.7, 56.3, 46.6, 18.4, 17.5, 13.7; HRESIMS *m/z* 233.0788 [M + Na]^+^ (calcd for C_11_H_14_O_4_Na: 233.0784). Compound **9:** ^1^H NMR (400 MHz, CDCl_3_), δ 6.45 (d, *J* = 2.4 Hz, 1H), 5.85 (d, *J* = 2.4 Hz, 1H), 5.02 (d, *J* = 8.2 Hz, 1H), 4.39 (dt, *J* = 6.4, 4.0 Hz, 1H), 3.52 (m, 1H), 3.16 (t, *J* = 6.4 Hz, 2H), 1.77 (m, 2H), 1.52 (m, 2H), 1.45 (m, 2H), 1.31 (m, 6H), 1.29 (m, 2H); ^13^C NMR (100 MHz, CDCl_3_), δ 169.7, 167.4, 134.6, 126.3, 85.1, 74.2, 62.9, 44.2, 36.1, 32.7, 29.3, 29.2, 29.0, 25.7, 24.8; HRESIMS *m/z* 305.1359 [M + Na]^+^ (calcd for C_15_H_22_O_5_Na: 305.1359).

### Cell culture and reagents

Madin-Darby canine kidney (MDCK) II cells were cultured in Dulbecco’s modified Eagle’s medium (Nacalai Tesque, Kyoto, Japan) supplemented with 10% fetal calf serum (Nichirei Biosciences, Tokyo) and 1% penicillin-streptomycin (Nacalai Tesque) in a humidified atmosphere containing 5% CO_2_.

Capsaicin, FD4, and NEM were purchased from Sigma (St. Louis, MO). LatA was from Wako Pure Chemical Industries (Osaka, Japan). DTT was from Nacalai Tesque, and A-967079 was from Cayman (Ann Arbor, MI).

Antibodies to occludin (#71-1500), Zo-1 (#61-7300), and tricellulin (#700191) were purchased from Invitrogen (Grand Island, NY). Anti-claudin-1 (#13050-1-AP) and anti-cofilin (#3312) was purchased from Proteintech Group, Inc. (Rosemont, IL) and Cell Signaling Technology (Beverly, MA), respectively. Anti-β-actin (#125K4769) and anti-phospho-cofilin (Ser 3, #sc-21867-R) were from Sigma and Santa Cruz Biotechnology (Santa Cruz, CA), respectively. Horseradish peroxidase (HRP)-conjugated anti-mouse and anti-rabbit IgGs were from Kirkegaard & Perry Laboratories (Gaithersburg, MD).

All other reagents were of reagent grade and purchased from Nacalai Tesque unless otherwise noted.

### Intracellular Ca^2+^ measurements

MDCK II cells were seeded in 96-well plates at a density of 3.4 × 10^4^ cells per well. The cells were cultured for 3 days, and the medium was changed every day to establish monolayer integrity. After specific treatments, monolayers were washed with Hank’s balanced salt solution (HBSS) and treated with fluo-8 loading buffer (5 μM Fluo-8 [#21080, AAT Bioquest, Sunnyvale, CA], 2.5 mM probenecid [#ALX-430-113-G005, Cosmo Bio, Tokyo], 1% F-127 [#59004, Biotium, Hayward, CA] in HBSS) at 37 °C for 1 h. Cells were washed with HBSS twice, and recording buffer (2.5 mM probenecid in HBSS) and various compounds were added. The Fluo-8 fluorescence was measured every 3 min for 60 min with a PowerscanHT fluorescence microplate reader (Dainippon Sumitomo Pharma, Osaka, Japan) at the excitation and emission wavelengths of 485 and 528 nm, respectively.

### Transport studies

We measured the MDCK II monolayer permeability using FD4 as described^15^.

In briefly, MDCK II cells were seeded in 6.5 mm diameter transwells (pore size: 0.4 μm, area: 0.33 cm^2^) coated with collagen, at a density of 3.4 × 10^4^ cells per well. The cells were cultured for 3 days to establish monolayer integrity. Transwell plates were washed three times, incubated with Hank’s balanced salt solution (HBSS) and equilibrated for 1 h at 37 °C. HBSS (0.1 ml) containing 1.0% w/v of FD4 was placed on the apical side and each transport enhancer was added to the apical side. The basolateral side was exposed to HBSS (0.6 ml), which was refreshed at predetermined intervals. Samples collected from the basolateral compartments were analyzed for FD4 using a PowerscanHT fluorescence microplate reader (Dainippon Sumitomo Pharma Co., Ltd., Osaka, Japan) at an excitation wavelength of 485 nm and an emission wavelength of 530 nm.

### TER measurements

For the TER experiments, MDCK cells were seeded in 12 mm-diameter transwells (pore size 0.4 mm, Corning Inc., Corning, NY) coated with collagen at a density of 1.1 × 10^5^ cells per well. The cells were cultured for 3 days to establish monolayer integrity. TER was measured as previously described^15^.

### Immunoblotting

MDCK II cells were seeded in poly-L-lysine-coated 24-well plates at a density of 2.1 × 10^5^ cells per well. The cells were cultured for 3 days, and the medium was changed every day to establish monolayer integrity. After specific treatments, monolayers were washed once with phosphate-buffered saline (PBS) and lysed with lysis buffer. After sonication, the cell extracts were boiled at 60 °C for 10 min, separated by sodium dodecyl sulfate-polyacrylamide gel electrophoresis (SDS-PAGE), transferred to a polyvinylidene fluoride microporous membrane (Millipore, Billerica, MA), blocked with 5% skimmed milk (Megmilk Snowbrand, Sapporo, Japan) or setsuyakukun supporter (#DRC-BS500CH, DRC, Tokyo), probed with the appropriate primary antibody and HRP-conjugated anti-IgG secondary antibody, and detected by enhanced chemiluminescence (#07880-70, Nakalai Tesque). Images were visualized using X-ray film (RU-X, Fuji film) for Figs 4A and 5E, and using Sayaka Imager (DRC, Tokyo) for Fig. 6E. The band intensities of phospho-cofilin, occludin and actin were measured with Image J and normalized by actin.

### Immunofluorescence staining

After specific treatments, MDCK II monolayers grown on LAB-TEK chamber Permanox slides (Nunc, Rochester, NY) were washed with PBS, fixed for 10 min with 3.7% formaldehyde, and permeabilized for 5 min in PBS containing 0.2% Triton X-100. Each antibody diluted in PBS containing 0.5% BSA was applied and incubated for 1 h at 37 °C. After washing with PBS, samples were stained with secondary antibodies as above. F-actin was labeled for 1 h with CF488-phalloidin (Biotium, Inc., Fremont, CA) diluted in PBS containing 0.5% BSA and washed with PBS. The coverslips were mounted with 5 µg/ml Hoechst 33258 (Sigma) in PBS containing 60% glycerol. Fluorescence images were acquired with a Leica DMI6000B microscope (Leica Microsystems, Heidelberg, Germany).

### Stable cell line with knockout of TRPA1

We established a stable MDCK II cell line with the knockout of TRPA1 using the CRISPR/Cas9 system. The non-viral all-in-one plasmid containing CRISPR Cas9 nuclease, sgRNA-targeted canine TRPA1, the neo gene for G418 selection, and mCherry as a fluorescent reporter were purchased from GeneCopoeia (Rockville, MD). Transfection was performed using Lipofectamine 3000 (Invitrogen) according to the manufacturer’s instructions. The isolation of transfectants was performed as described^14^. We sequenced the PCR products of the modified clones in order to detect the indel mutations.

### Data analysis

We calculated the theoretical clog *P*-values with ChemDraw Pro ver. 13.0.2.3021 software (PerkinElmer, Waltham, MA). We used GraphPad Prizm 6 to perform Tukey’s multiple comparison test for Fig. 1F,G, and Student’s *t*-test or Dunnett’s test for Fig. 4B to obtain the *p-*values.

## Electronic supplementary material

Supplementary Information
