# Supplementary material for: TRPA1-dependent reversible opening of tight junction by natural compounds with an α,β-unsaturated moiety and capsaicin
Source: Sci Rep. 2018 Feb 2;8:2251. doi: 10.1038/s41598-018-20526-7 (PMC5797179; doi:10.1038/s41598-018-20526-7)
Supplement: Supplementary file 1 — Supplementary Information [file 41598_2018_20526_MOESM1_ESM.docx]

**TRPA1-dependent reversible opening of tight junction by natural compounds with an α,β-unsaturated moiety and capsaicin**

Yusuke Kanda^1^, Youhei Yamasaki^1^, Yoshie Sasaki-Yamaguchi^2^, Noriko Ida-Koga^2^, Shinji Kamisuki^2,3^, Fumio Sugawara^2^, Yoko Nagumo^4^, and Takeo Usui^4,*^

^1^Graduate School of Life and Environmental Sciences, and ^4^Faculty of Life and Environmental Sciences, University of Tsukuba, 1-1-1 Tennodai, Tsukuba, Ibaraki 305-8572, Japan

^2^Department of Applied Biological Science, Faculty of Science and Technology, Tokyo University of Science, 2641 Yamazaki, Noda, Chiba 278-8510, Japan

^3^School of Veterinary Medicine, Azabu University, 1–17–71 Fuchinobe, Chuo-ku, Sagamihara, Kanagawa 252–5201, Japan

*Corresponding author: Takeo Usui

Faculty of Life and Environmental Sciences, University of Tsukuba

1-1-1 Tennodai, Tsukuba, Ibaraki 305-8572, Japan

Tel: +81-29-853-6629 Fax: +81-29-853-4605

E-mail: [usui.takeo.kb@u.tsukuba.ac.jp](mailto:usui.takeo.kb@u.tsukuba.ac.jp)

This file includes Supplementary Table S1, and Figure S1-S11.

Supplementary Table S1. Structure-activity relationships

|  | **Reactive moiety and related functional group** | **cLog*P* value** | **FD4 permeability (reversibility)** | **Requirement of TRPA1** |
| --- | --- | --- | --- | --- |
| **Capsaicin (1)** |  | 4.20 | Yes (reversible) | Yes |
| **Pyrenocine A (2)** | α,β-unsaturated ketone | -0.58 | Yes (reversible) | Yes |
| **Pyrenocine H (3)** | α,β-unsaturated ketone | -0.58 | Yes (partial) | N.T. |
| **Compound 6** | Ketone (no conjugated double bond) | -0.56 | No |  |
| **Pyrenocine C (7)** | α,β-unsaturated, hydroxyl group | -0.57 | No |  |
| **Dehydrocurvularin (4)** | α,β-unsaturated ketone (*endo*) | 2.03 | Yes (partial) | N.T. |
| **Curvularin (8)** | Ketone (no conjugated double bond) | 2.05 | No |  |
| **Avenociolide (5)** | α,β-unsaturated ester (*exo*) | 3.00 | Yes (partial) | N.T. |
| **Compound 9** | α,β-unsaturated ester (*exo*) | 1.80 | No |  |
| **NEM** | Maleimide | 0.12 | Yes (partial) | N.T. |


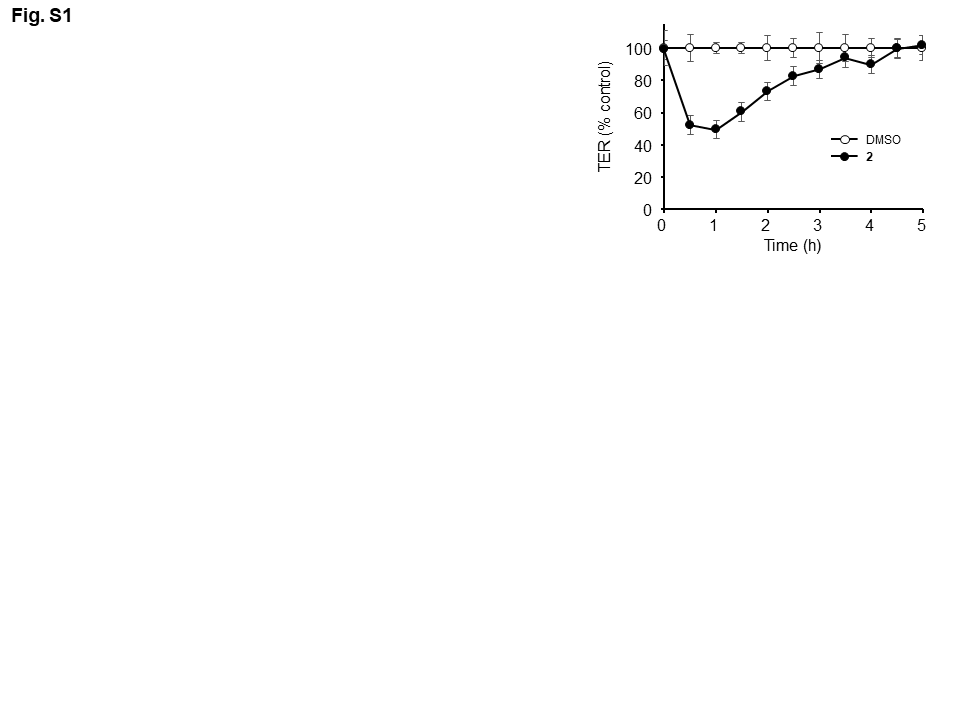


**Supplementary Figure S1. Compound 2 decrease TJ permeability transiently in the MDCKII cell monolayer.**

TER measurement in MDCKII monolayer. ○: DMSO, ●: 30 μM compound **2**. Values represent mean ± S.D. Typical data of three independent experiments is shown.


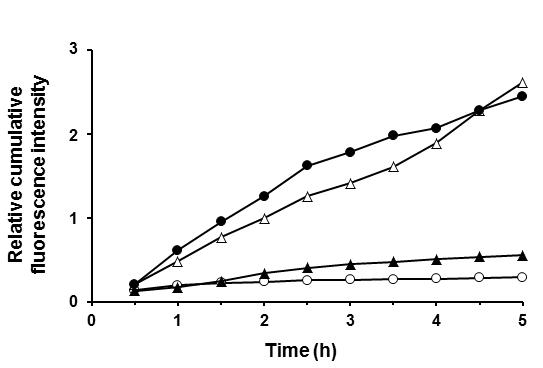


**Supplementary Figure S2.** **Structural analog lacking α,β-unsaturated ketone failed to increase TJ permeability in the MDCKII cell monolayer.**

Compound **8**, an analog of compound **4** with only difference in α and β position saturated, hardly enhanced the permeability compared to compound **4**. ○: DMSO, △: 0.1 μM latrunculin A, ●: 10 μM compound **4**, and ▲: 10 μM compounds **8**. Typical data of three independent experiments is shown.


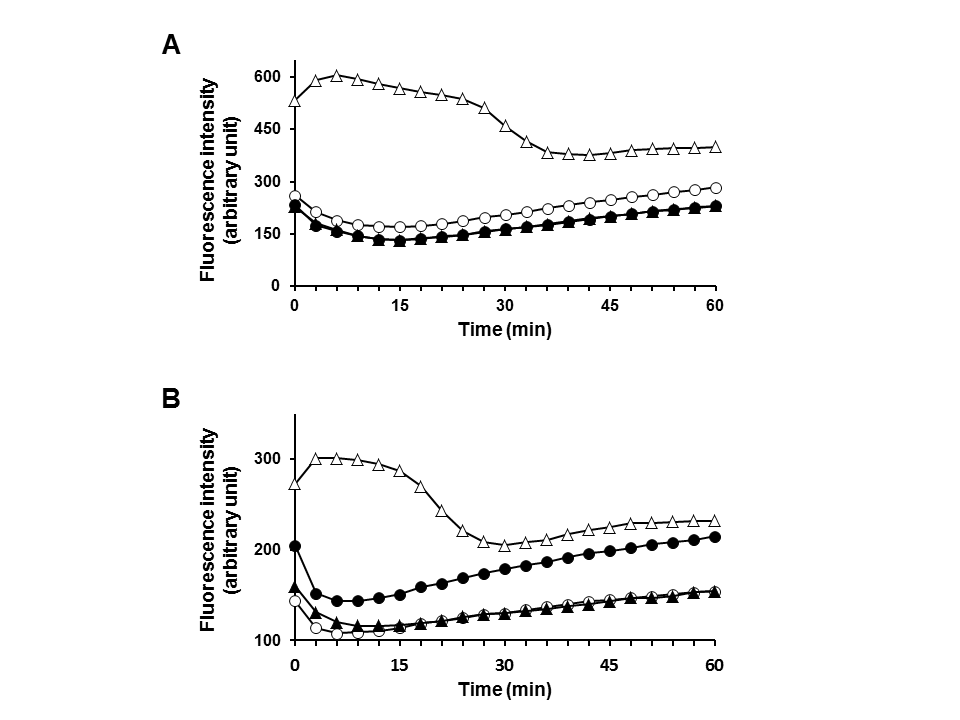


**Supplementary Figure S3. Structural analogs without TJ opening ability failed to induce Ca^2+^ influx.**

**A.** Compound **6** and **7** did not induce Ca^2+^ influx in MDCKII monolayer. ○: DMSO, △: 10 μM ionomycin, ●: 30 μM compound **6**, ▲: 30 μM compound **7**. Typical data of three independent experiments is shown.

**B.** Compound **8** and **9** did not induce Ca^2+^ influx in MDCKII monolayer. ○: DMSO, △: 10 μM ionomycin, ●: 10 μM compound **8**, ▲: 10 μM compound **9**. Typical data of three independent experiments is shown.


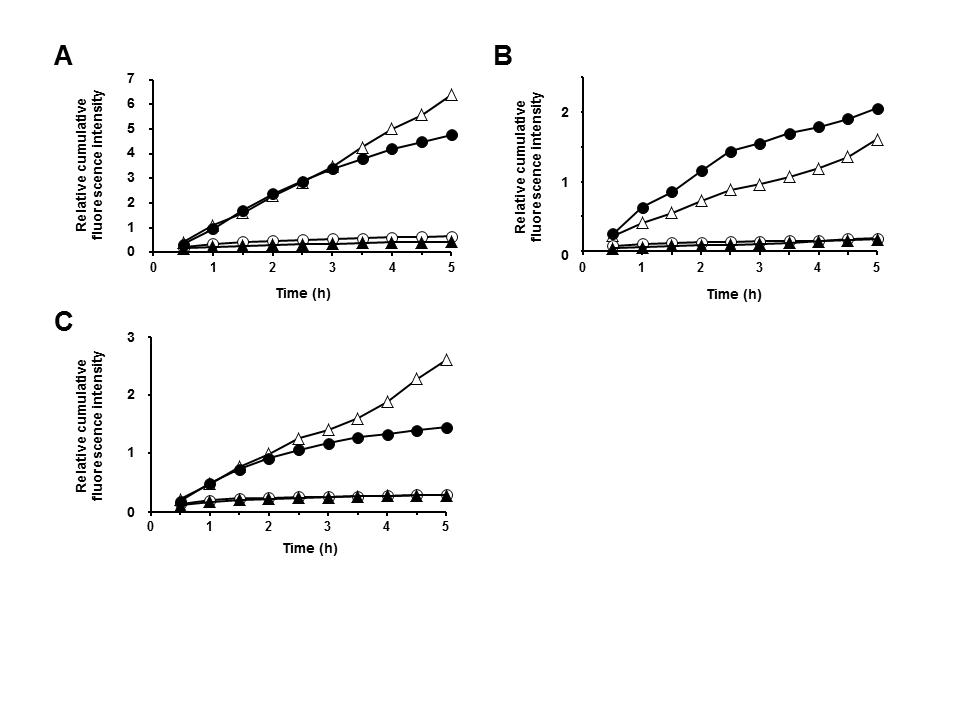


**Supplementary Figure S4. Compound 3, 4 and 5 lost its ability to open TJ by pretreatment with DTT.**

**A.** Pretreatment of DTT abolished the TJ opening ability of compound **3**. ○: DMSO, △: 0.1 μM latrunculin A, ●: 30 μM compound **3**, ▲: 30 μM compound **3** pretreated with 30 μM DTT.

**B.** Pretreatment of DTT abolished the TJ opening ability of compound **4**. ○: DMSO, △: 0.1 μM latrunculin A, ●: 10 μM compound **4**, ▲: 10 μM compound **4** pretreated with 10 μM DTT. Typical data of three independent experiments is shown.

**C.** Pretreatment of DTT abolished the TJ opening ability of compound **5**. ○: DMSO, △: 0.1 μM latrunculin A, ●: 10 μM compound **5**, ▲: 10 μM compound **5** pretreated with 10 μM DTT. Typical data of three independent experiments is shown.


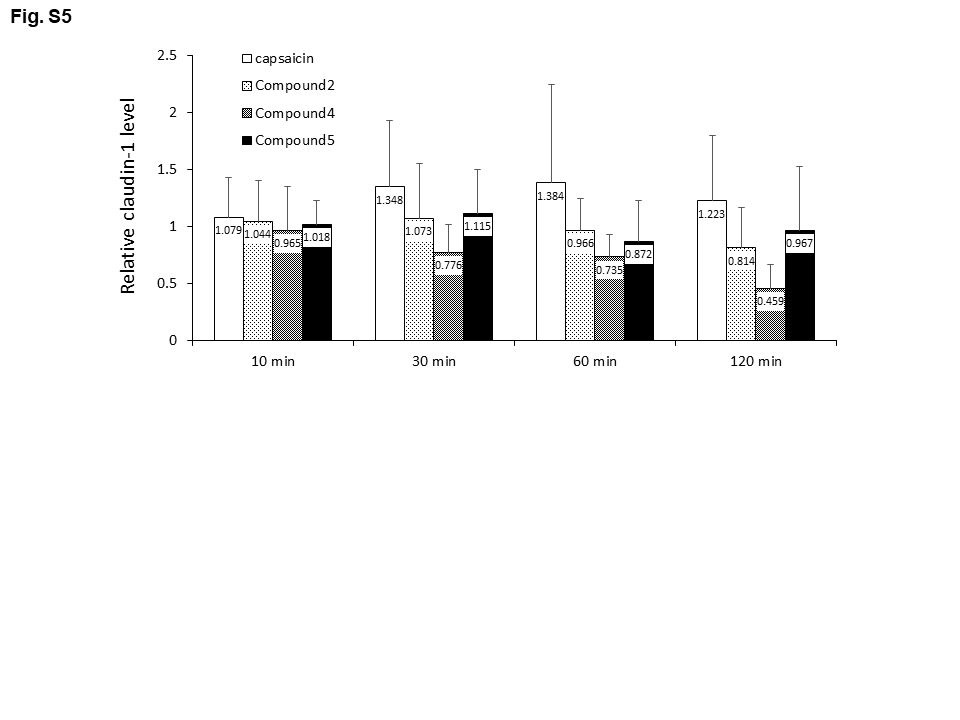


**Supplementary Figure S5. Claudin-1 shows no apparent change by compound 2, 4 and 5.**

The densitometric analysis of claudin-1 was performed with NIH ImageJ software. Mean ± SD is shown for capsaicin (n = 11), compound **2** (n = 6), compound **4** (n = 4), and compound **5** (n = 4).

**
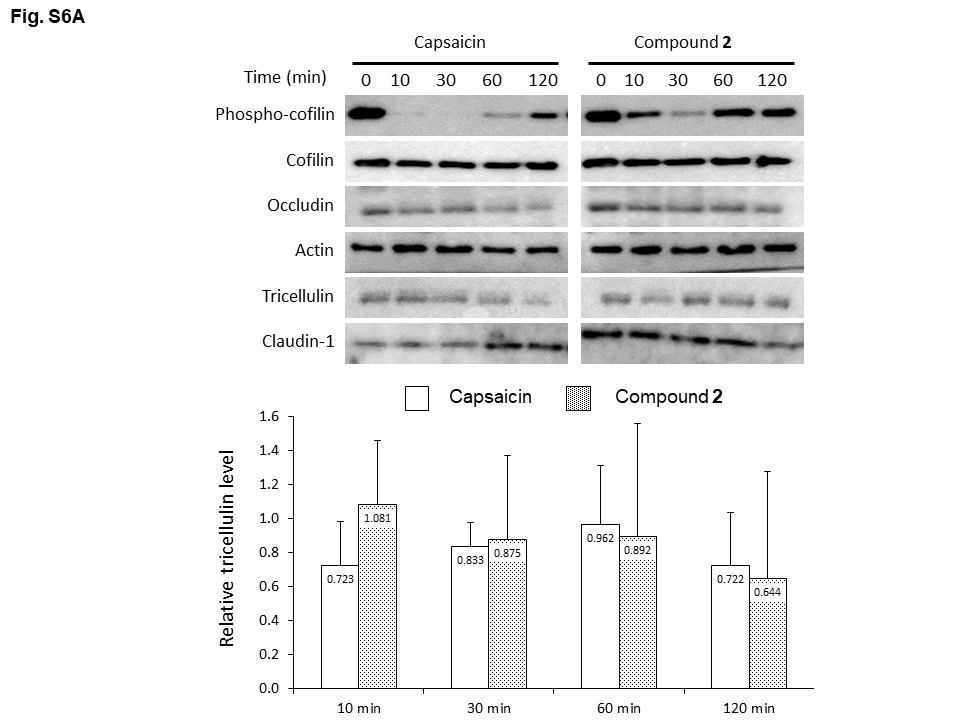
**

**
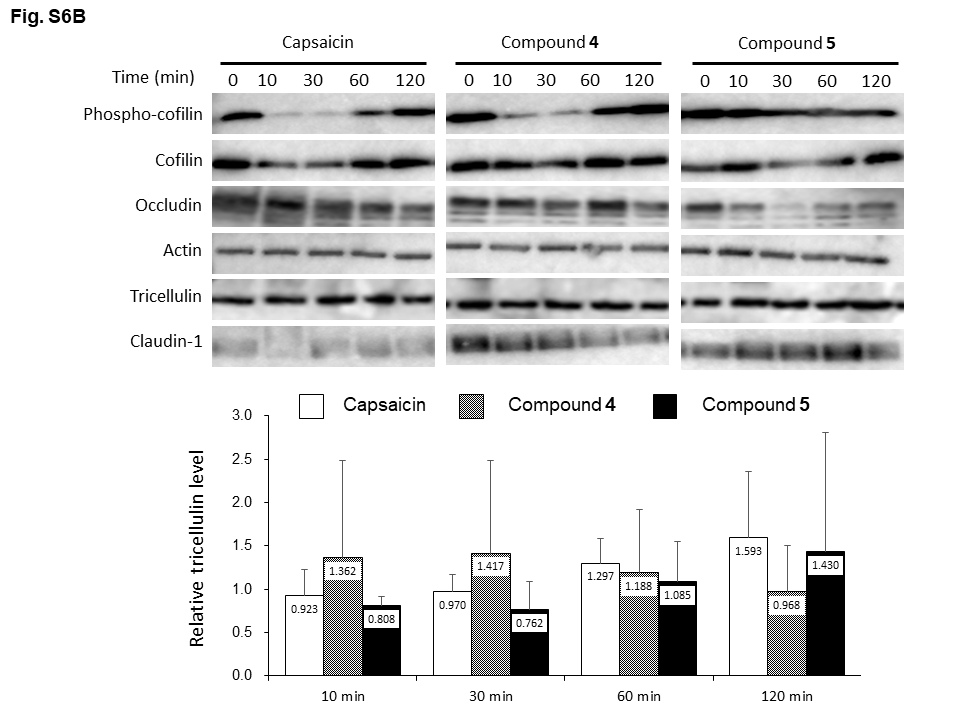
**

**Supplementary Figure S6. Effects of compounds 2, 4, and 5 on the amount of tricellulin.**

MDCKII monolayers were treated with DMSO, 30 μM compounds **2, 4, and 5** for indicated time. The densitometric analysis of tricelluin was performed with NIH ImageJ software. Mean ± SD from three independent experiments is shown. The band intensity of each time point relative to that of time 0 are shown. Student's t-test for compound **2** and Dunnett’s test for compounds **4** and **5** has been performed comparing each compound with capsaicin at each time point.

**
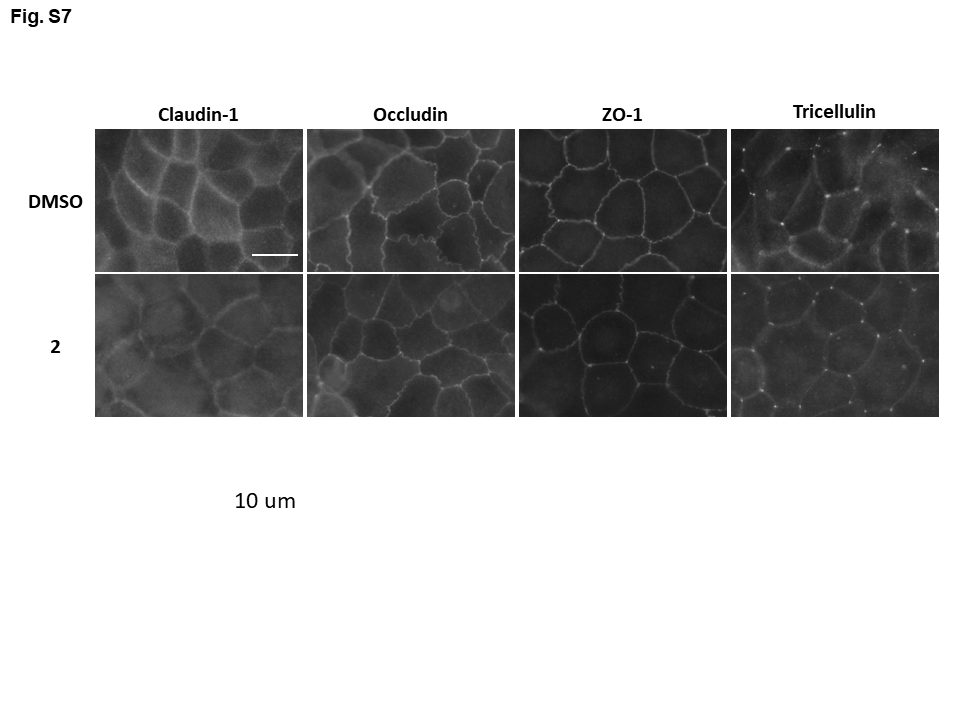
**

**Supplementary Figure S7. Effects of compound** 2 **on the distribution of TJ proteins.**

MDCKII monolayers were treated with DMSO, 30 μM compound **2** for 45 min and were stained with each TJ antibodies (claudin-1, occludin, Zo-1, and tricellulin). Typical data of three independent experiments is shown. Scale bar = 10 μm.


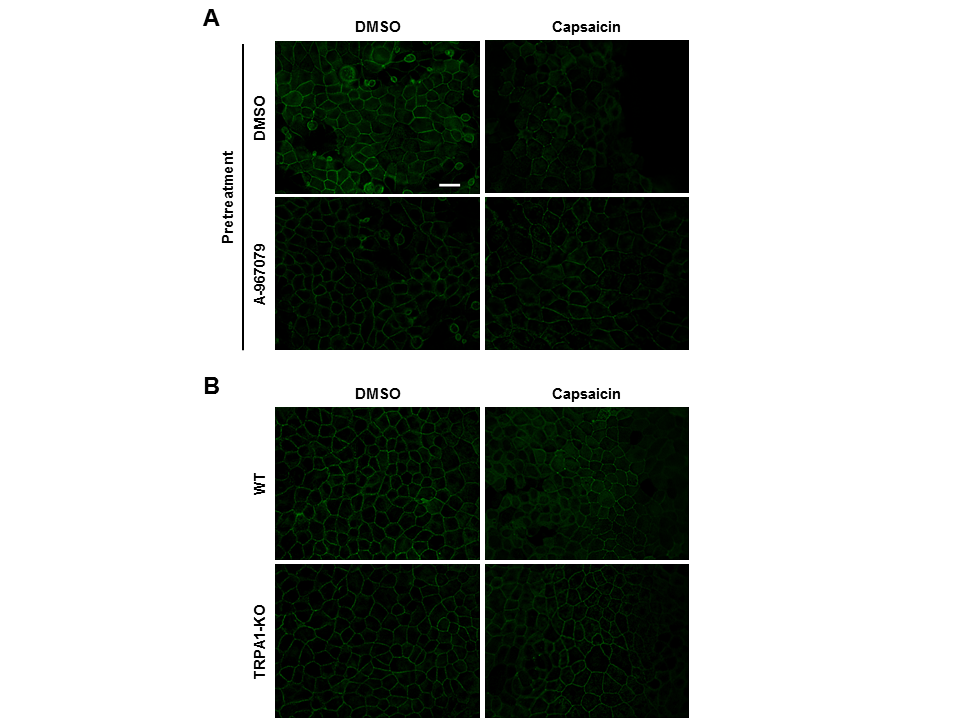


**Supplementary Figure S8. TRPA1 involved in the capsaicin-induced actin reorganization.**

**A.** Pretreatment of TRPA1 antagonist attenuated the actin reorganization induced by 300 μM capsaicin. WT cells were treated with or without 1 μM A-967079 for 30 min, followed by 300 μM capsaicin treatment for 45 min. Typical data of three independent experiments is shown. Bar: 20 μm.

**B.** The actin reorganization induced by capsaicin was attenuated in TRPA1-KO cells compared to WT cells. WT and TRPA1-KO cells treated with 300 μM capsaicin treated for 30 min. Typical data of three independent experiments is shown. Bar: 20 μm.


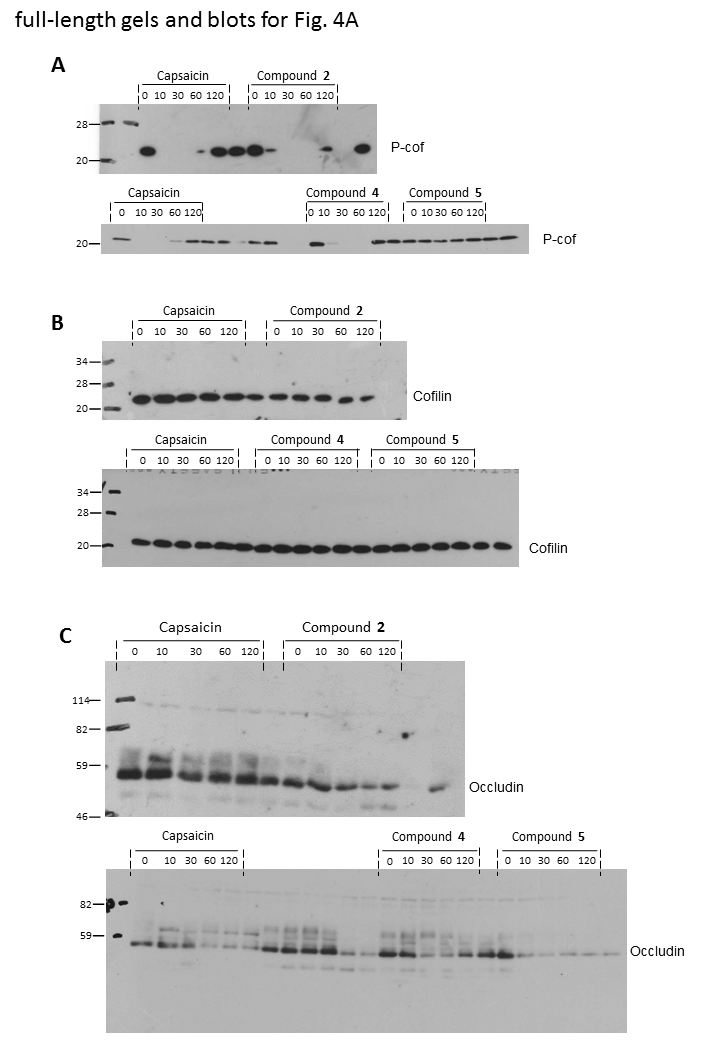

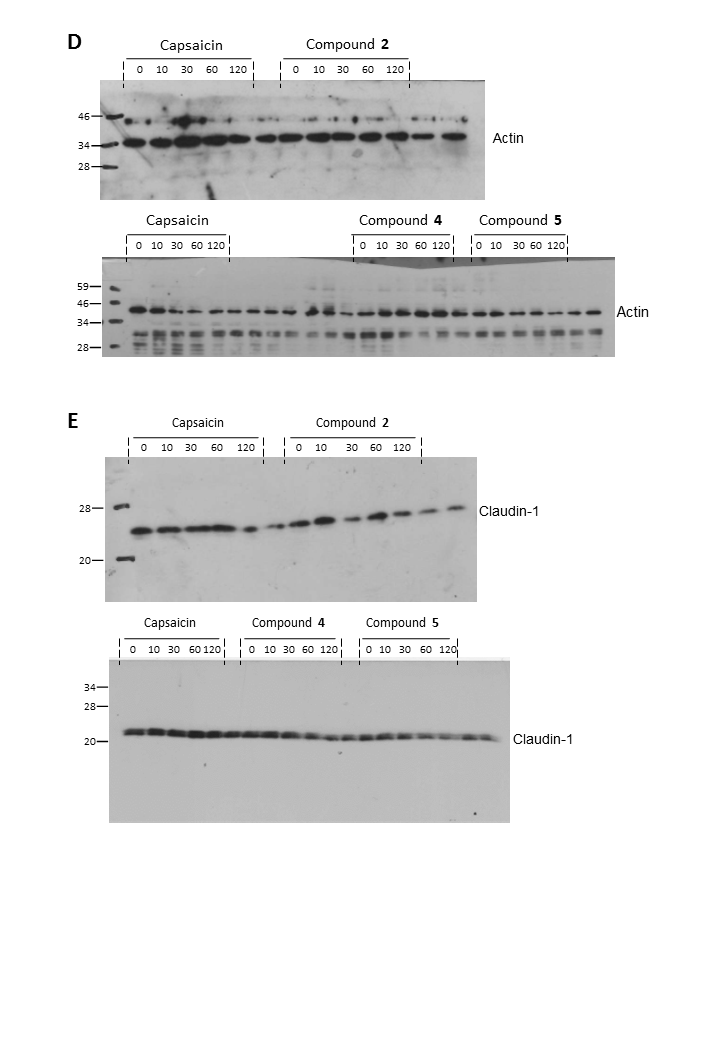


**Supplementary Figure S9. Original full-length blot of Figure 4A**

**A.** Phosphorylated cofilin, **B.** Cofilin, **C.** Occludin, **D.** Actin, and **E.** Claudin-1


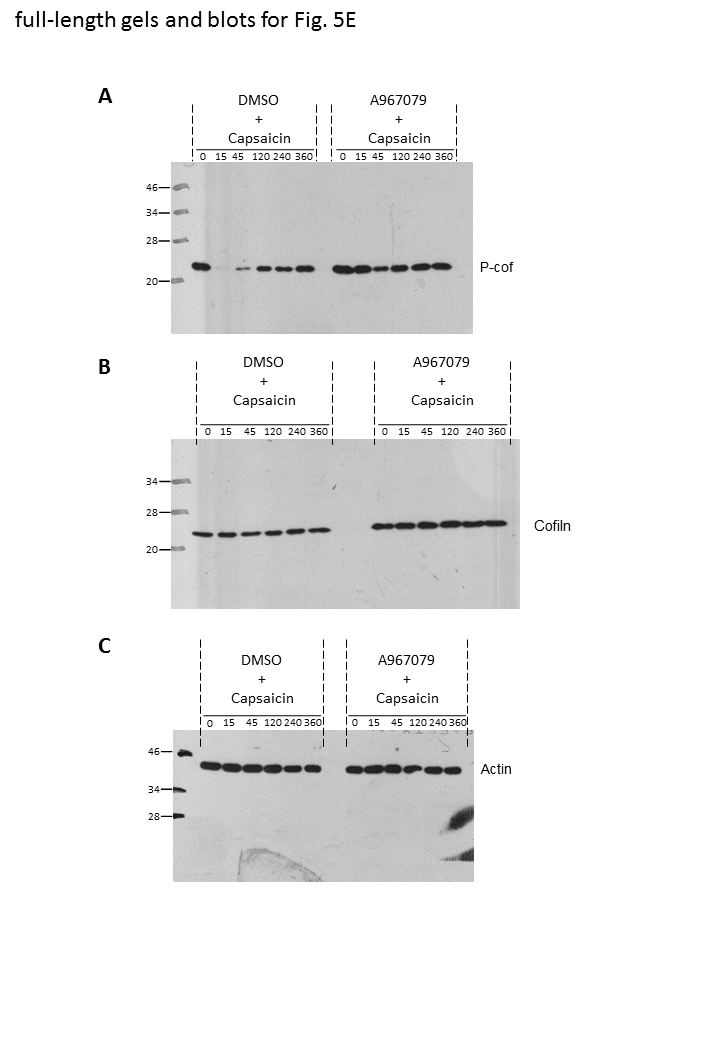


**Supplementary Figure S10. Original full-length blot of Figure 5E**

**A.** Phosphorylated cofilin, **B.** Cofilin, and **C.** Actin


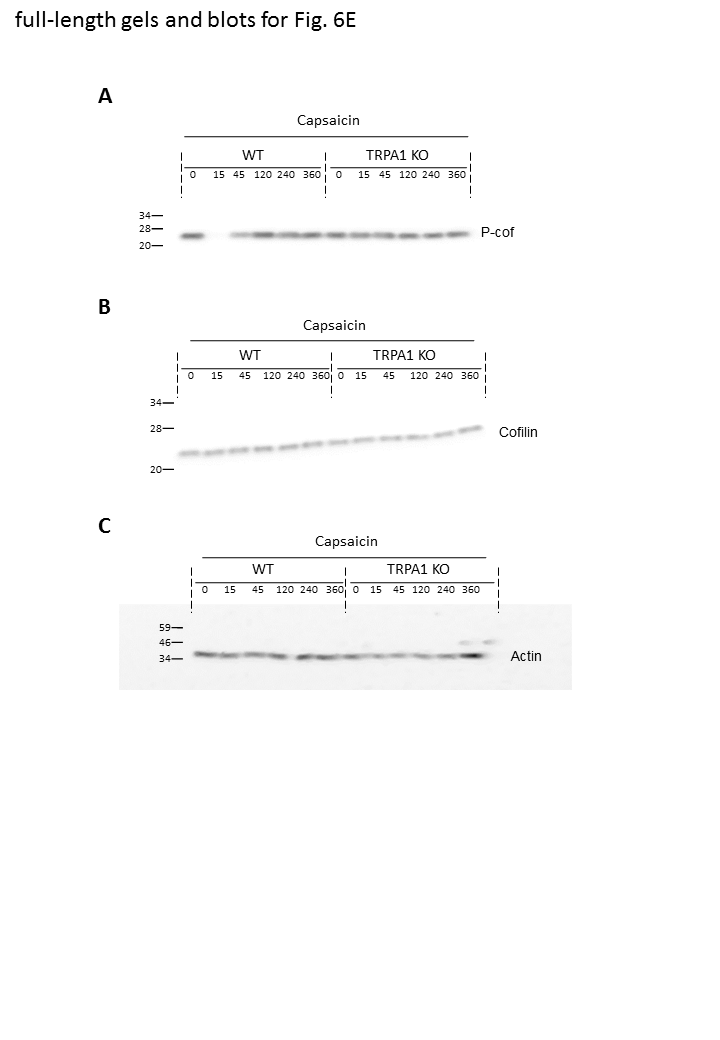


**Supplementary Figure S11. Original full-length blot of Figure 6E**

**A.** Phosphorylated cofilin, **B.** Cofilin, and **C.** Actin
